# Recent Development of Neural Microelectrodes with Dual-Mode Detection

**DOI:** 10.3390/bios13010059

**Published:** 2022-12-30

**Authors:** Meng Xu, Yuewu Zhao, Guanghui Xu, Yuehu Zhang, Shengkai Sun, Yan Sun, Jine Wang, Renjun Pei

**Affiliations:** 1 CAS Key Laboratory for Nano-Bio Interface, Division of Nano-biomedicine, Suzhou Institute of Nano-Tech and Nano-Bionics (SINANO), Chinese Academy of Sciences, Suzhou 215123, China; 2 School of Nano-Tech and Nano-Bionics, University of Science and Technology of China (USTC), Hefei 230026, China

**Keywords:** neural microelectrodes, dual-mode, electrophysiological signal, in vivo electrochemical sensing, neurotransmitter, brain-computer interface (BCI)

## Abstract

Neurons communicate through complex chemical and electrophysiological signal patterns to develop a tight information network. A physiological or pathological event cannot be explained by signal communication mode. Therefore, dual-mode electrodes can simultaneously monitor the chemical and electrophysiological signals in the brain. They have been invented as an essential tool for brain science research and brain-computer interface (BCI) to obtain more important information and capture the characteristics of the neural network. Electrochemical sensors are the most popular methods for monitoring neurochemical levels in vivo. They are combined with neural microelectrodes to record neural electrical activity. They simultaneously detect the neurochemical and electrical activity of neurons in vivo using high spatial and temporal resolutions. This paper systematically reviews the latest development of neural microelectrodes depending on electrode materials for simultaneous in vivo electrochemical sensing and electrophysiological signal recording. This includes carbon-based microelectrodes, silicon-based microelectrode arrays (MEAs), and ceramic-based MEAs, focusing on the latest progress since 2018. In addition, the structure and interface design of various types of neural microelectrodes have been comprehensively described and compared. This could be the key to simultaneously detecting electrochemical and electrophysiological signals.

## 1. Introduction

Neurons are the basic structure and functional units of the nervous system, having functions of sensing stimuli and conducting excitations [1]. Neurons communicate via complex patterns of electrical and chemical signals [2]. Electrical signals are the ion concentration change on both sides of the neuron cell membrane, leading to the rapid potential change in nerve fibers [3]. Chemical communication is the process in which neurotransmitters (NTs) and other neurochemicals are released into the synaptic gap from a nerve cell. It is usually from the synaptic vesicles enriched in presynaptic cells, and then binds to the receptors on the targeted postsynaptic cells [4]. Chemical signals are obtained by monitoring the changes in the neurochemical levels. Neurons utilize these two communication patterns to maintain the operation of each region along with the interconnection of the entire neural network. The generation and conduction of electrical activities in normal brain physiological events are often regular and limited. When pathological events occur in the brain, neurons are likely to have abnormal discharges such as epilepsy. Therefore, deep brain stimulation (DBS) electrodes can regulate abnormal neural electrical activity in the target area, advancing as an effective neural regulation technology [5]. In addition, pathological events inside the brain are often accompanied by impaired neurotransmission, such as synaptic plasticity defects and synaptic loss. These can manifest in abnormal neurotransmitters (NTs) and/or other neurochemicals levels in the brain. The lack of abnormality of various neurotransmitters can cause multiple neurodegenerative diseases, such as epilepsy, Parkinson’s disease, and Alzheimer’s disease. Moreover, cognitive decline occurs due to cholinergic and glutamate deficiency. Additionally, there is a lack of synaptic plasticity, inhibitory and excitatory neurotransmitter homeostasis disorders within epileptic symptoms, neuropsychiatric monoamine neurotransmission, etc. [6,7,8].

We need to synchronously detect the electrophysiological and chemical signals in the brain to decipher the mechanism of various physiological and pathological events behind the complex interconnection of neural networks. With the development of the brain–computer interface (BCI), different neural electrodes can detect the electrophysiological activities of single or multiple neurons [9,10,11,12,13]. An interesting research direction is their amperometric combination with in vivo electrochemical sensors to obtain dual-mode detection neural electrodes [14,15]. The primary advantage of amperometry is that it can directly determine a number of molecules by calculating peak area [16,17,18]. Neurons communicate with each other in both electrical and chemical aspects. Therefore, a high spatial and temporal resolution of electrical and chemical dual-mode analysis technology can facilitate a better understanding of brain function. Moreover, it can highlight many potential mechanisms of some nervous system diseases. Thus, the brain regions of interest can be associated with behavioral variables to complement the information. Single detection mode has been studied extensively [19]. For example, many experiments have improved the electrochemical detection electrode of carbon fiber in vivo. Therefore, it is a better electrochemical detection electrode [20,21,22]. It is essential to obtain the interactive and cooperative information between the two modes and understand the brain operation mechanism. Thus, some researchers have begun researching and developing new dual-mode electrodes to detect electrochemical and electrophysiological signals in vivo simultaneously. In 2008, Barbosa et al., evaluated the available strategies for simultaneous electrochemical and electrophysiological measurements in the brain using microelectrodes and MEAs, particularly ceramic-based MEAs [23]. Mao et al., demonstrated the electrochemical biosensor development for neurochemicals in vivo, especially enzyme electrochemical biosensors [24]. This paper systematically reviews the latest progress of neural microelectrodes depending on electrode materials for in vivo electrochemical sensing and electrophysiological signal recording, included carbon-based microelectrodes, silicon-based MEAs, and ceramic-based MEAs, the latest progress since 2018. In addition, the structures and materials for different neural microelectrodes were comprehensively described and differentiated.

## 2. Electrochemical Detection

Electrochemical technology can detect neurochemical substances. It differs from traditional spectrophotometry, electrophoresis, liquid chromatography, and fluorescence detection methods. Electrochemical technology can achieve accurate, rapid, and real-time detection in vivo. Electrochemical detection utilizes microelectrodes or microprobes to record the current generated by various neurochemicals at the corresponding oxidation peak potential, thereby achieving chemical signal detection. It has high sensitivity, fast response, and a high signal-to-noise ratio (SNR) [25,26,27].

The potentiostatic amperometric method is minimally affected by the change of capacitance current. Thus, it has superior time resolution and high sensitivity but has poor selectivity. It is used for detecting known neurotransmitters in single cells. Different selective coatings can be applied to block the interference factors on the electrode recording site. Thus, it allows specific molecules to pass through the coatings and conduct electrochemical detection on the electrode surface. For instance, coating Nafion can repel anionic interactions, enhance in vivo selectivity, and lead to interference and noise subtracted and recognized [28]. Cyclic voltammetry (CV) synthesizes different analyte responses along the potential axis. It is dependent on their electrochemical properties, leading to selectivity in the measurement [29]. Therefore, fast-scan cyclic voltammetry (FSCV) can also be applied with a high resolution of sub-second (hundreds of milliseconds). Its combination with carbon fiber electrodes enables high resolution and low tissue damage detection of neurotransmitters. However, FSCV is not entirely applicable to all the scenes. When multiple target analytes detected simultaneously possess similar oxidation potential during a high scanning rate, it is challenging to distinguish the results. In addition, a high scanning rate can generate a sizeable capacitive current, which should be subtracted from CV to determine a small Faraday current due to the oxidation or reduction of the target analyte. Elisa Castagnola et al., confirmed that the voltage reduction and oxidation peak of dopamine (DA) and 5-hydroxytryptamine (5-HT) are distinguished by employing optimal FSCV triangular waveform, having scan rates ≤ 700 V s^−1^ with potentials holding and shifting at 0.4 V and 1 V [30]. Differential pulse voltammetry (DPV) combines the square wave technique with linear sweep voltammetry. At a constant frequency, it employs a small amplitude square wave (~25 mV) as a signal superimposing the slow linear potential slope. The charging current is strongly discriminated, and the ratio of faradaic to charging current is very large. Therefore, DPV is a voltammetry technology with high sensitivity. The differential DPV current has a symmetrical volt-ampere peak, whose intensity is directly proportional to the analyte concentration. Thus, it can synchronously detect the oxidation potential of two compounds differing by more than 100 mV without interfering with each other. Moreover, the selectivity of sensing specific substances is also optimized [31]. The complex brain environment requires that microelectrodes have high sensitivity and the ability to resist interference effects. Electrodes should be selective, or people should distinguish the signals of various neurochemical substances via different strategies to detect various neurochemical substances. In addition, most of the existing electrochemical detection electrodes can only be used for acute detection. Additional electrode design is required for chronic detection with high time resolution and long-term stability in vivo [32].

## 3. Electrophysiological Signal Detection

**Figure 1:**
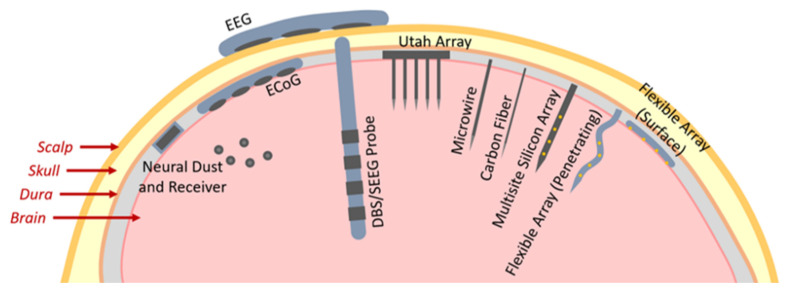
The major types of neural electrode interfaces in brain [33].

**Figure 2:**
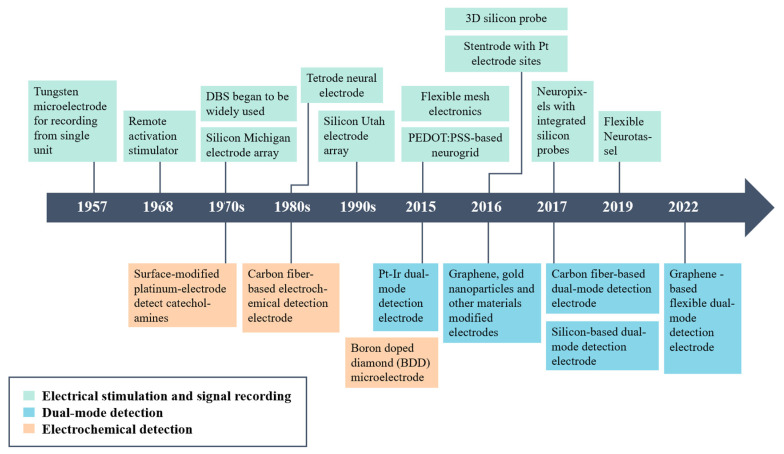
The development trend of various electrodes is indicated on the time axis [35,36,38,39,40,41,42,43,44,45,46].

Three types of neural electrodes are used to capture the electrophysiological activities of neurons: non-invasive, semi-invasive and invasive. The non-invasive electrode is usually a head-worn type, does not require surgery but has a poor spatial resolution, and can only detect electroencephalography (EEG) signals. The semi-invasive electrode is implanted between the skull and the cerebral cortex. It causes less tissue damage than the invasive electrode, thereby measuring the electrocorticography (ECoG) signal. The major types of neural electrode interfaces in brain are shown in Figure 1. The implanted nerve electrode should pass through the cerebral cortex and enter the brain tissue. Therefore, there is a significant requirement for the safety of the electrode and the surgical process. It requires implantable electrodes of high spatial and temporal resolution for a single neuron to record and modulate neural activity in a sub-millisecond [33]. The implantable electrodes were gradually developed from single-channel to multi-channel recording from the metal microwire electrode [34] at the beginning to the Utah and Michigan electrodes [10,35,36] based on silicon. It had excellent performance detecting electrophysiological signals or controlling neural activities, including the deep brain stimulation (DBS) electrode mentioned above for clinical treatment of psychomotor disorders. Moreover, it can capture local field potentials (LFPs) through external cables connected to constantly developing DBS conductors [37]. The development trend of various types of electrodes on the time axis is in Figure 2.

The electrode to detect neural electrical activity should have excellent electrochemical properties, including high charge storage capacity and low electrode impedance. The effective area of electrode sites can be increased by modifying the electrode surface to decrease the impedance [47]. The electrode surface can also be modified using various materials to achieve high-quality signal acquisition. These materials include metal or metal compound materials such as platinum black [48], iridium oxide [49], and titanium nitride; carbon materials such as carbon fibers and carbon nanotubes [50]; conductive polymer materials such as PEDOT and other composite materials [51]. Many of them can improve biocompatibility while enhancing electrochemical performance. Anti-inflammation coating [52] and neurotrophic factors coating [53] can also elevate biocompatibility. For example, researchers studied a new electrode analog of the cochlear implant, polydimethylsiloxane (PDMS) filaments, to reduce any inflammation caused by the implant. It contains anti-inflammation/fibrosis dexamethasone (Dex) and is coated using hyaluronic acid (HA) as the surface modifier. The PDMS filaments were prepared by mixing Dex into PDMS containing poloxamer 188 (P188) in varying amounts as a drug release enhancer. The results indicated that the filaments containing 5% Dex, 5% P188, and HA coatings were significantly reduced by 51% in the fibroblast cell number. Moreover, the surface cell adhesion was significantly decreased [54]. The elastic modulus of traditional nerve implant materials, such as silicon and metal, is higher than the elastic modulus of brain tissue (silicon and metal range from 50 to 200 Gpa, while nervous tissues are 3.15–10 kPa) [55]. Long-term implantation of brain tissue will lead to continuous electrode cutting due to brain tissue micromotion. Thus, it displaces the electrode interface, neurons, and glial scar [56]. These will damage the body and affect the signal recording quality. Therefore, more flexible implantable electrodes have been developed. Some are material-based electrodes composed of biocompatible polymers such as polyimide and parylene [57]. Others are modified with a layer of flexible materials, including hydrogel, on the original electrode surface [58]. Flexible electrode materials have lower bending stiffness than rigid electrode materials, leading to better mechanical compliance. The implanted flexible electrode generates little shear movement as the bending stiffness is closer to the nerve tissue, thereby reducing the chronic immune response. Gilberto Filho et al., designed the 3D-printed molds to fabricate a fully polymeric electrode depending on PEDOT:PSS:DMSO. The polymer-based electrode has a mechanical strength similar to the brain. The conductive ink depending on PEDOT: PSS: DMSO has a conductivity of 137 S/cm and a resistance of 180.7 ± 19.5 Ω. Therefore, the immune response of the full polymeric electrode is completed after 21 days of implantation. Thus, there is no significant change in the recorded signal quality. In addition, 3D printing technology makes flexible electrode manufacturing more accessible and faster [59]. Many experiments have confirmed that these flexible materials can significantly elevate the flexibility of electrodes, thereby enhancing their biocompatibility, decreasing biological reactions, and enhancing long-term stable measurements of electrodes in vivo [60,61,62].

Many electrodes based on different materials have been developed. Table 1 summarizes the overall characteristics of these electrode materials.

## 4. Dual-Mode Neural Microelectrodes

### 4.1. Carbon-Based Neural Microelectrodes

Carbon is an attractive chronic implant material to minimize tissue damage [79]. This is because of its chemical inertness, biocompatibility, good electrical performance, electrochemical stability, pure capacitive charge injection (no irreversible reactions and byproducts), and rapid surface electrochemical kinetics. Carbon is often used as electrode materials, including carbon fiber, carbon nanotube, glassy carbon, graphene, etc. The electrochemical performance can be improved because of the porous channels connected inside the carbon to enable the rapid migration of electrons and ions. Therefore, carbon is a promising implantable neural electrode material for electrophysiological and electrochemical dual-mode detection.

#### 4.1.1. Carbon Fiber Microelectrodes (CFEs)

The diameter of carbon fibers utilized for neural microelectrodes is less than 10 μm. It is suitable for implantation and causes less tissue damage than traditional electrodes. Carbon fiber microelectrode is a valuable tool for in vivo detection of neurotransmitters. This is because of its small size, high sensitivity, biocompatibility, and good electrochemical properties [80,81]. Mao et al., have done much work and made some progress in the in situ electrochemical detections of carbon fiber microelectrodes. They utilized the as-synthesized vertically aligned carbon nanotube-sheathed carbon fibers (VACNT-CFs) as the microelectrode to detect ascorbate acid (AA) in vivo. Thus, it has high reproducibility and selectivity. Microelectrodes with original VACNT-CFs electrode material are synthesized by assembling VACNT-CFs into capillaries. Carbon nanotubes (CNTs) can significantly promote AA oxidation (ca. −50 mV) at low potential, opening up a new way for selective AA detection. Experiments indicate that the oxidation potential of AA is well separated from the oxidation potential of other electrochemical active substances. Therefore, the developed electrode has fast electron transfer kinetics for AA electrochemical oxidation. Even if other electrically active substances (e.g., dopamine and 5-hydroxytryptamine) coexist in the rat brain, they can also be used for highly selective and repetitive real-time AA monitoring [82]. They also used platinized vertically aligned carbon nanotube (VACNT)-sheathed carbon fibers (Pt/VACNT-CFs) as the electrodes to detect the dynamic change of O_2_ in vivo. The VACNT-CFs are developed by the pyrolysis of iron phthalocyanine (FePc) on the surface of CFs, then through electrochemical deposition of platinum nanoparticles to synthesize Pt/VACNT-CFs. Platinum (Pt) is the most active metal for the electrochemical reduction of O_2_ because it facilitates O_2_ removal via a four-electron process to produce water. CNTs are heterogeneous porous Pt catalyst scaffolds and can prevent electrochemical dissolution and separation. The microelectrode designed and manufactured by combining VACNT CFs with Pt demonstrates a new method for monitoring O_2_ in vivo without forming toxic H_2_O_2_ intermediates [83]. Mao et al., have developed various strategies to improve the electrode to inhibit the adsorption of biomolecules in the brain on the implanted microelectrode surface during electrochemical detection. These include designing the polymer monomer EDOT-PC (amphoteric choline phosphate functionalized ethylene dioxythiophene) and polymerizing it on the microelectrode surface via electrochemical polymerization. This forms a PEDOT-PC ultrathin film using a cell membrane-like structure. A thin PEDOT-PC film is formed due to the self-limitation of electrochemical polymerization at the PC end, which ensures the rapid mass transfer of the substance for film detection. Therefore, PEDOT-PC modified microelectrode can effectively resist the non-specific adsorption of proteins and maintain the detection sensitivity of the electrode. PEDOT-PC modified CFE was utilized to accurately monitor DA release during KCl stimulation and electrical stimulation in the rat brain. Another example of avoiding the non-specific adsorption of proteins on the electrode surface is designing the covering CFE with leukocyte membranes (LMs). Leukocytes facilitate immune function in the body and can promote the immune evasion of nanoparticles. We found that these decorated CFEs controlled their electrochemical reactivity and indicated significant resistance to non-specific protein adsorption by layering LMs on the surface of CFE, thus extending the life of implanted CFEs [84]. During the in situ analysis, solving the critical problem of protein adsorption of microelectrodes in vivo through various strategies will build the foundation for deciphering the molecular mechanism of brain neurochemistry.

Many strategies have also emerged to elevate the selectivity of microelectrodes for neurochemicals. Aptamers are short, synthetic single-stranded nucleic acids, specifically identifying multiple targets with high affinity. The combination of molecular recognition properties of aptamers with implantable electrochemical platforms will enhance selectivity for molecular detection. Using a positively charged coating, pretreating the electrode surface can load the aptamer to the CFE surface by electrostatic interaction. However, this binding is easily destroyed by an ionic effect and exhibits low stability in the physiological environment. Mao et al., have demonstrated a new surface functionalization strategy. This assembles the aptamer cholesterol amphiphilic molecule over the alkyl chain functionalized CFE. The aptamer can be effectively fixed on the CFE surface with the help of the non-covalent cholesterol alkyl chain interaction. The results indicate that this strategy greatly improves the selectivity of DA detection in rat brains. Compared with the bare carbon fiber electrode, the modified electrode selectivity to DA is increased three times [85]. Enzymatic modification of microelectrodes can improve the selectivity of microelectrodes to neurochemicals. Matias Regiart et al., developed a highly selective and sensitive nanostructure biosensor to simultaneously determine lactic acid and glucose in rat brains. It was based on carbon fiber microelectrode (CFM) modified by nanoporous gold (NPG) with the dynamic hydrogen bubble template (DHBT) method. Platinum nanoparticles (PtNPs) electrodeposited on NPG films can enhance the sensitivity of H_2_O_2_ detection and electrocatalytic performance. The nanostructure microelectrode platform was modified using immobilizing glucose (GOx) and lactate (LOx) oxidases. Therefore, the electrode has a high sensitivity to H_2_O_2_ (5.96 A M^−1^ cm^−2^) at 0.36 V vs. Ag/AgCl. The linear range was from 0.2 to 200 μM, and the LOD was 10 nM. Moreover, the basic extracellular concentrations of lactic acid and glucose were also determined in vivo [86].

In electrophysiological detection, a carbon fiber electrode array (CFEA) becomes a substitute for metal wire or silicon probe. The carbon fibers are thinner and more flexible than commonly used metal wires and silicon, with a lower immune response after implantation. Grigori Guitchounts et al., designed the 64-channel CFEA and the batch preparation method of recording sites. The tip was prepared using sulfuric acid etching to enhance the surface area and was modified with PEDOT-TFB. This led to the same tip impedance reducing from 4.84 ± 0.68 to 0.17 ± 0.86 MΩ. The recording in the cortex of rats establishes the feasibility of recording neural signals with this method [87].

**Figure 3:**
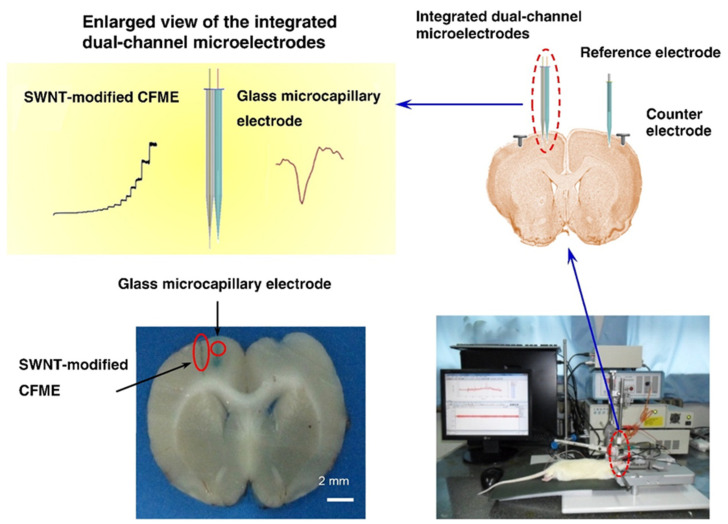
The schematic diagram of the experimental setup for simultaneous monitoring of AA and electrophysiological activity in vivo.

Depending on the excellent performance of carbon fiber microelectrode to detect neurochemical substances in vivo, Mao et al., integrated the carbon fiber microelectrode with the electrophysiological detection electrode to synthesize an integrated dual-mode microelectrode (IDMME). This supports real-time recording of AA and electrical signals in vivo. The electrochemical detection electrode has been manufactured from carbon fiber modified with carbon nanotubes. Moreover, the glass microcapillary electrode is manufactured from fiber-filled borosilicate glass tubing, having an inner diameter of 0.68 mm and an outer diameter of 1.5 mm. It is used as the electrophysiological recording channel for single-unit brain recording. The experimental setup diagram is depicted in Figure 3. These two independent technologies do not interfere. The experiment demonstrates that the current has a linear relation with AA concentration within the 0 to 1200 μM range (γ = 0.972). The investigation also explored that the amperometric method did not produce residual artifacts on adjacent electrophysiological records. Thus, the feasibility of IDMMEs to selectively monitor the level of AA and single-unit electrical signals is established in vivo. The final results revealed that the increase of cortical ascorbate level in the early stage of ischemia was parallel to the significant reduction of single unit activity. The inverse changes in ascorbate level and single unit activity designate a complex neurochemical process during the acute phase of global cerebral ischemia. Additionally, the increase in AA and the decrease in neural activity can be induced by brain acidosis, hypoxia depolarization, and several injuries after global cerebral ischemia/perfusion [88]. 

**Figure 4:**
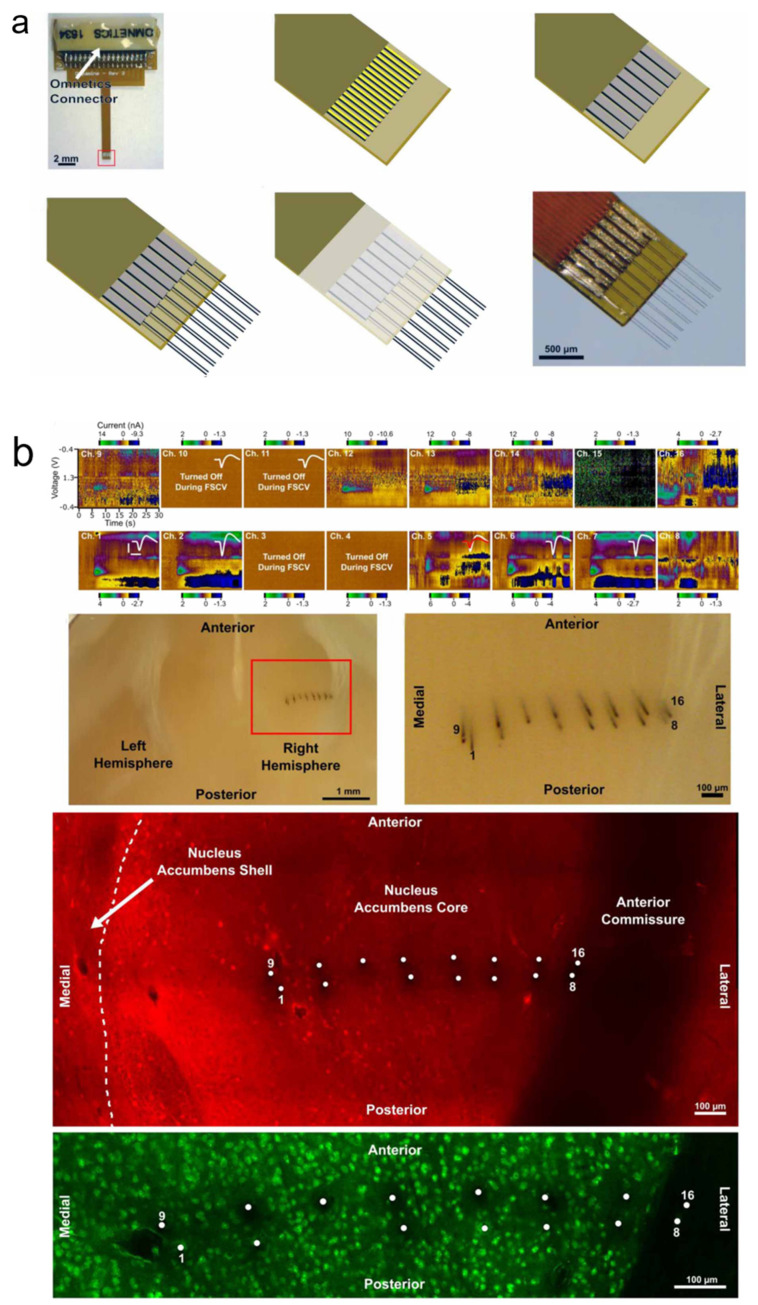
(**a**) The manufacturing process of a flexible array. (**b**) Chronic detection of AA and electrophysiology signals, carbon fiber anatomical localization, and immunohistological reaction.

Patel et al., fabricated a 16-channel array electrode using a carbon fiber substrate and coated Parylene C. In addition to being an insulating layer, its good flexibility also reduces the electrode’s footprint, thereby increasing the biocompatibility of the electrode interface. The detection sites of electrophysiology and electrochemistry use the same electrode material. The manufacturing process of the flexible array is displayed in Figure 4a. We used laser ablation for selective re-exposure on the carbon fiber surface for functionalization. The experimenter explored the impedance and in vitro dopamine detection experiments from the carbon fiber array electrode. This helped optimize tip treatment conditions of 50 μm in length, thereby plasma graying the probe. Then, the electrode array was implanted into the rat nucleus accumbens for one month to detect chronic electrophysiology and DA signaling. Experimental results showed DA release on eleven channels in vivo, and on the same day, unit activity was detected on seven channels. The entire array was sliced 78 days after implantation without any significant movement of the electrodes. The histological experiment revealed minimal tissue damage (Figure 4b), and we quantified the density of neurons around the electrodes. The results indicated that the density within the first 100 μm was almost indistinguishable from a normal brain after more than 10 weeks of implantation [89].

#### 4.1.2. Graphene-Based Microelectrodes

Graphene-based nanomaterials are utilized in many microelectrode designs due to their high conductivity, excellent flexibility, and biocompatibility, thus forming a stable electrode–nerve interface [90,91,92]. In addition, the optical transparency of the graphene interface enables neural electrodes to have a multimodal approach. Moreover, the electric layer is compatible with other microfluidic or optical manipulation ports. These multi-modalities can provide a next-generation interface for neural network research with high-fidelity activity patterns. Farida Veliev et al., performed in vitro detection of spontaneous hippocampal neuron activity using a millimeter-size PDMS fluid chamber based on in situ grown graphene sensors. Various experiments have established the reliability of detecting neural activity [82]. Bao et al., developed a flexible, stretchable neurochemical biosensor based on NeuroString. They embedded a laser-induced graphene nanofiber network into an elastomer matrix. The NeuroString sensor can detect the dynamics of multiple neurotransmitters in the brain and gut in real time. The sensor has a high level of flexibility and stretchability similar to tissue, thereby maintaining the distinctive electrochemical properties of nanomaterials [93]. Due to the excellent properties of graphene, it can be used to design dual-mode detection microelectrodes for electrophysiology and electrochemistry in vivo.

#### 4.1.3. Glassy Carbon (GC) Microelectrodes

**Figure 5:**
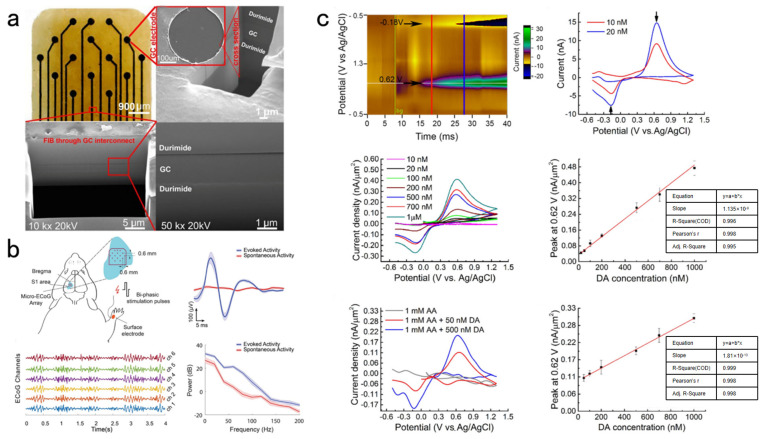
(**a**) The details of the GC neural probe. (**b**) Sensory evoked potentials were induced through the bi-phasic stimulation pulses on the right wrist. The ECoG microelectrode array was used to record them. (**c**) In vitro dopamine detection.

Surabhi Nimbalkar et al., proposed a glassy carbon microelectrode with capacitive behavior. It can sustain over 3.5 billion bi-phasic pulse cycles at a charge density of 0.25 mC/cm^2^, with a high charge storage capacity (CSC). These probes can maintain stability to avoid long-range electrical stimulation corrosion by applying a novel two-step double-sided mode transfer method with GC structure. GC nerve probes are fabricated from a homogeneous material and encapsulated on the flexible film polyimide substrate. Therefore, the excellent electrochemical stability of GC materials was utilized, which improved the biocompatibility of film devices. The novel fabrication is not involved with the intermediate metal deposition process. These probes have a high signal-to-noise ratio (>16) of electrical signal recording and real-time high-resolution neurotransmitter detection within the same platform. It was shown by FIB cross-section characterization and SEM images that the GC microstructure had strong adhesion to the top insulating layer and the bottom substrate layer with that of the hydroxyl and carbonyl covalent bonds. This is confirmed by extensive in vivo and in vitro experiments based on the highest reported CSC (61.4 ± 6.9 mC/cm^2^) and high-resolution DA detection at 10 nM levels within uncoated neural probes [79]. Figure 5 depicts the GC neural probe and sensory evoked potentials caused by the bi-phasic stimulation pulses. These were recorded by the ECoG microelectrode array and in vitro dopamine detection.

Elisa Castagnola et al. [94] developed a 4-channel intracortical glassy carbon (GC) MEA over a flexible substrate to detect neural activity and dopamine at four different brain locations. The microfabrication technology is ameliorated by an extra augment layer to enable brain penetration. For example, a thicker layer of polyimide was coated on the insulation layer to improve the penetration of the device. Genki Ogata et al., proposed a drug-tracking system composed of a glassy microelectrode and a microsensor. The microsensor was made of boron-doped diamond for tracking pharmacokinetics and detecting the neuronal local field potentials in the rat brain [95].

#### 4.1.4. Diamond Microelectrodes

**Figure 6:**
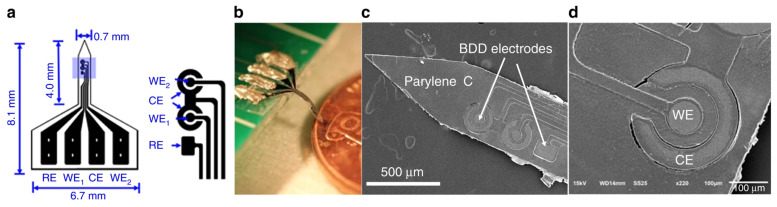
(**a**) Diagram of the BDD microelectrode probe. (**b**,**c**) Picture and SEM images of the manufactured neural probe. (**d**) A close-up view of the BDD WE and CE have been provided by the SEM image.

MEAs made entirely of diamond with the single material microelectromechanical system concept (SMM) have successfully been fabricated [96]. They completed the electrophysiological and electrochemical experiments in vivo and in vitro, respectively. Chan et al., designed a novel polycrystalline diamond (poly-C)-based microprobe using an undoped poly-C as its support material. Young’s modulus was in the compatible range of 400–1000 GPa. The poly-C resistivity of boron-doped was about 10^−3^ Ω·cm. Thus, it was utilized as an electrode material to provide a stable interface for chemical and electrical signal detection for neural research. The probe has eight poly-C electrode detection sites with a 2~150 μm diameter, and the electrode capacitance was approximately 87 μF/cm^2^. The minimum detectable concentration of norepinephrine is about 10 nM. It has been implanted into the auditory cortex region of guinea pig brains for in vivo neural studies, with a recording signal amplitude of 30–40 μV and a 1 ms duration [97]. Diamond has good biocompatibility, chemical inertness, low double-layer capacitance, and other characteristics. However, its high hardness is not conducive to being used as an implant to a certain extent. Fan et al., demonstrate a pliable microelectrode probe fabricated of a diamond. The microelectrode comprises a polycrystalline boron-doped diamond (BDD) probe supported on a flexible Parylene C substrate through multiple channels (Figure 6). A wafer manufacturing method is developed and ameliorated for utilizing the growth side of the BDD thin film instead of the nucleation side as the sensing surface. In comparison, the growth side had a lower background current and broader water potential window [88]. In addition, the modification of nanodiamond to carbon fiber electrodes to enhance the electrochemical performance and electrochemical sensing of the electrode has been proposed by Maryam A. Hejazi et al. [15]. The researchers developed a new method for preseeding carbon fibers using covalently bound nanodiamonds before diamond growth to protect carbon fibers during chemical vapor deposition. The covalent bonding of nanodiamonds is realized by grafting aromatic amines to connect nanodiamonds with carbon fiber surfaces. Thus, it reduces the difficulty of coating diamonds on carbon fibers in the past. This composite microelectrode can record the action potential of individual neurons, delivering effective electrical stimulation pulses and providing good dopamine electrochemical detection ability.

### 4.2. Silicon-Based Microelectrode Array

**Figure 7:**
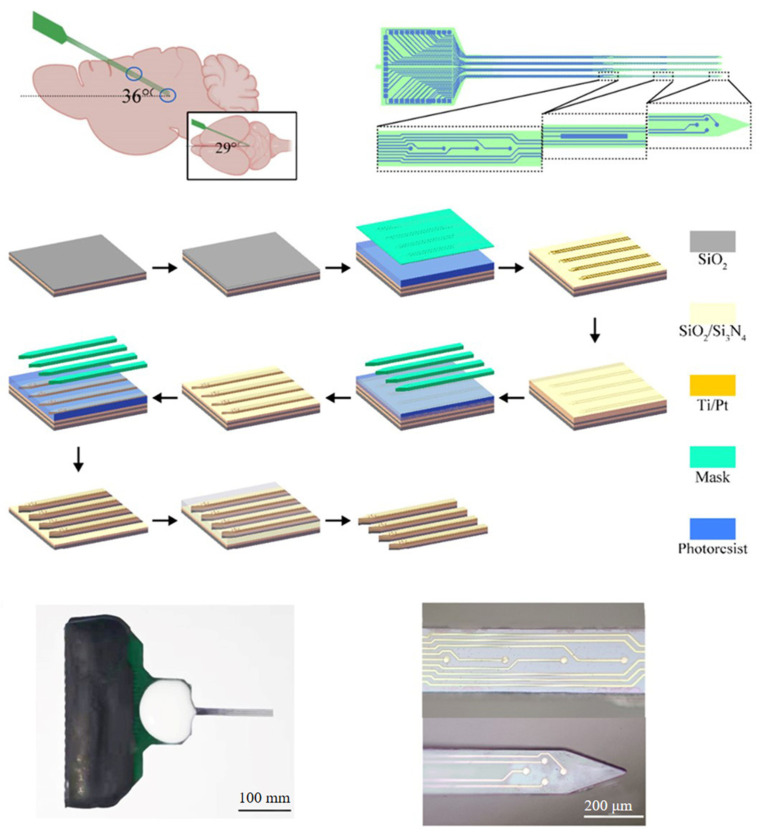
The design and fabrication of MEA.

In the 1970s, Wise et al., reported that the first silicon-based microprobes were fabricated on a rigid silicon substrate with lithography. It could precisely control the electrode spacing to 10 to 20 µm or larger. Moreover, the diameter of the electrode tip could be as small as 2 µm [36]. Silicon microprobe electrodes have excellent processability of silicon. Thus, silicon-based micromachining technology has emerged as one of the main tools for manufacturing neural MEAs using microscale accuracy and high reproducibility. Cai et al., developed a microelectrode array whose detection site arrangement matched the shape and position of rat dorsal periaqueductal gray (dPAG) through the microelectromechanical system (MEMS) technology. The detection performance was ameliorated by depositing platinum-black NPs. It could detect the electrophysiological signal of dPAG of pre- and post-activity neurons for free-behaving rats exposed to 2-methyl-2-thiazoline (2-MT), an effective analog of predator odor [98]. Cai et al., designed an MEA to study 5-HT deficiency-induced insomnia on the dorsal rap nucleus (DRN) and hippocampus neurons in rats. This enabled the simultaneous detection of DRN and hippocampus electrophysiological activities at a long distance [99]. Figure 7 depicts the design and fabrication of MEA.

Another common way to form a needle tip array through MEMS technology is on the silicon substrate. The tip is coated with platinum-black, iridium oxide, or other materials to conduct electricity. The electrode column is insulated using a Parylene C layer with good biocompatibility or directly developing the Parylene into a flexible microelectrode array [100,101]. Silicon microarray electrodes can fulfill the needs of most electrophysiological records and have been successfully deployed in many neuroscience applications [102,103]. In addition, silicon can be used as the sensor matrix material for neurochemical signals in the brain. Thus, silicon microprobe is used to realize dual-mode detection of electrophysiology and electrochemistry. Electrical and neurochemical activities can be correlated by using the same equipment [104]. M.D. Johnson et al. [105] developed a neural probe based on a Michigan silicon-substrate probe. It was formed on silicon using a planar process for simultaneously detecting neurochemical and electrophysiological signals in rats. The array had a single handle with seven platinum and seven iridium microelectrode sites. The platinum sites on each array were plated with platinum-black and electropolymerized with Nafion. It increases dopamine sensitivity by 74%, reduces the sensitivity of common interfering substances by at least 89%, and monitors neural activity within adjacent iridium sites. 

**Figure 8:**
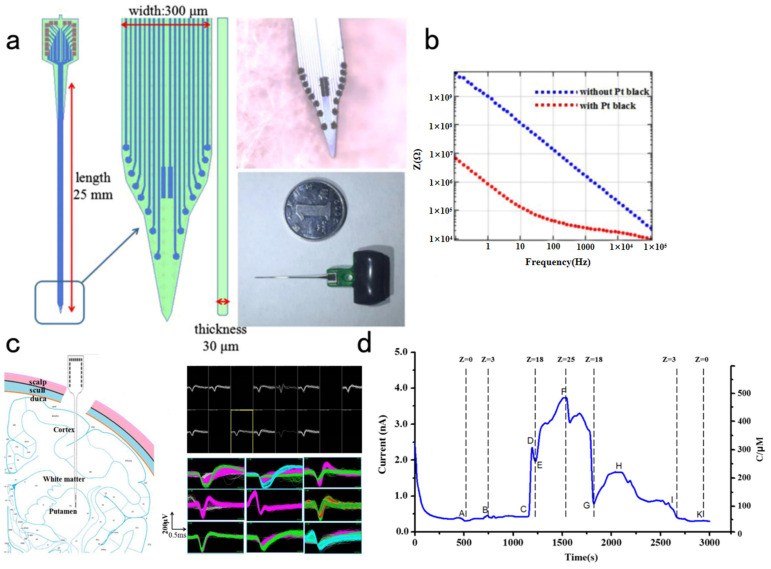
(**a**) The MEA probe is fabricated on silicon-on-insulator substrates through MEMS. (**b**) The average impedance is down from 1.52 to 0.026 MΩ at 1 kHz. (**c**) The MEA probe was attached to the recording system to detect Spikes and LFPs. (**d**) Ch7 was used to record the amperometric I-T graph during the sequential insertion and retrieval of the neural probe.

Furthermore, neural probes for non-human primates have been designed as brain research tools. Cai et al. [106] developed a low-cost silicon-based 16-bit implantable MEA chip by standard lithography technology for in vivo testing. The array was 25 mm long (Figure 8a). The ion exchange resin Nafion was coated dropwise on the probe tip to increase the selectivity of DA detection of platinum sites. Moreover, platinum-black nanoparticles were plated onto the bare microelectrode to lower impedance and enhance the ratio of signal to noise. Then, continuous high-quality electrophysiological and electrochemical signals were determined in different regions from the monkey’s cortex to the striatum. Compared with 1.52 MΩ before modification, the average microelectrode impedance decreased to 0.026 MΩ at 1 kHz (Figure 8b). The implanted MEA microelectrode was attached to the electrophysiological recording system. The spikes, LFPs, and currents were recorded during the sequential insertion and retrieval of the probe (Figure 8c,d). It has been possible to acquire high-quality dual-mode signals in monkey brains. However, it is still cumbersome and inefficient to implement because a combination of electrodes and commercial instruments is involved in the experiment. The acquisition of both signals cannot be completed using a single device. Therefore, Cai et al. [107] developed an integrated system for synchronous monitoring of nerve spikes and DA activity inside non-human primate brains. The system integrates implantable sensors, dual-function heads, and low-noise detection instruments. Then, they performed synchronized recordings of electrophysiological signals and DA in monkeys. The result indicated that the system typically had an input impedance level of 5.1 GΩ, an input-referred noise level of only about 3 μV_RMS,_ and a DA detection sensitivity of 14.075 pA/μM. Therefore, it could detect electrophysiological signals and DA without interfering with each other.

**Figure 9:**
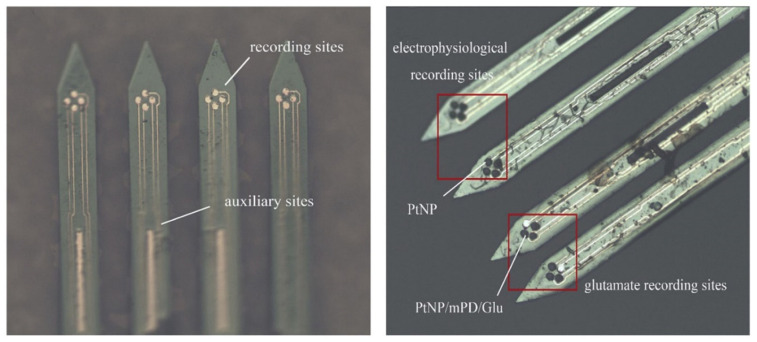
The electrode structure and site distribution.

The excellent properties of silicon-based MEAs provide new means for mechanism research and disease treatment [108]. Some studies have demonstrated that the disorder of excitatory glutamate-mediated neurotransmitters could be the primary cause of temporal lobe epilepsy (TLE). Cai et al. [109] designed a silicon-based MEA for simultaneously detecting neuroelectrophysiology and the neurotransmitter glutamate (Glu). They modified the electrophysiological recording site using platinum nanoparticles (PtNP) to reduce impedance and improve the SNR. Moreover, they also changed the glutamate oxidase enzyme (Gluox) by glutaraldehyde crosslinking at the glutamate detection site and plated m-phenylenediamine (mPD). The electrode structure and site distribution are demonstrated in Figure 9. Glutamate is oxidized and releases H_2_O_2_ under the catalysis of Gluox. mPD membranes effectively restrain the diffusion of AA, DA and 5-HT to the inner layers but permit H_2_O_2_ to penetrate. After it spreads to the PtNP layer, the H_2_O_2_ molecule is oxidized at the optimum voltage (0.6 V), releasing two electrons. The detected electrochemical current can reveal the glutamate concentration around the neuron since the electronic number is directly proportional to the glutamic molecular weight. The dual-mode MEA was implanted into the hippocampus of anesthetized TLE and normal rats. This helped investigate the spatial and temporal characteristics of glutamate efflux in the hippocampus Cornu Ammon 1 (CA1) of TLE rat seizures, non-seizures, and the differences in neural activity between TLE and normal rats. The experimental results indicated that the MEA probe showed excellent electrical performance (resistance is 14.2 ± 1.3 kΩ, SNR ≥ 4), sensitivity (6.276 ± 0.102 pA/μM), linearity (R = 0.9986) and selectivity (97.82%) while detecting glutamate in brain extracellular fluid. Simultaneously, the researchers observed that the nerve peak discharge during the seizure was denser and more regular than before. The amplitude of LFPs increased nearly three times, and the discharge power during the seizure changed more strongly. Glutamate concentration elevated with the increase of the neuron discharge frequency and LFP power.

**Figure 10:**
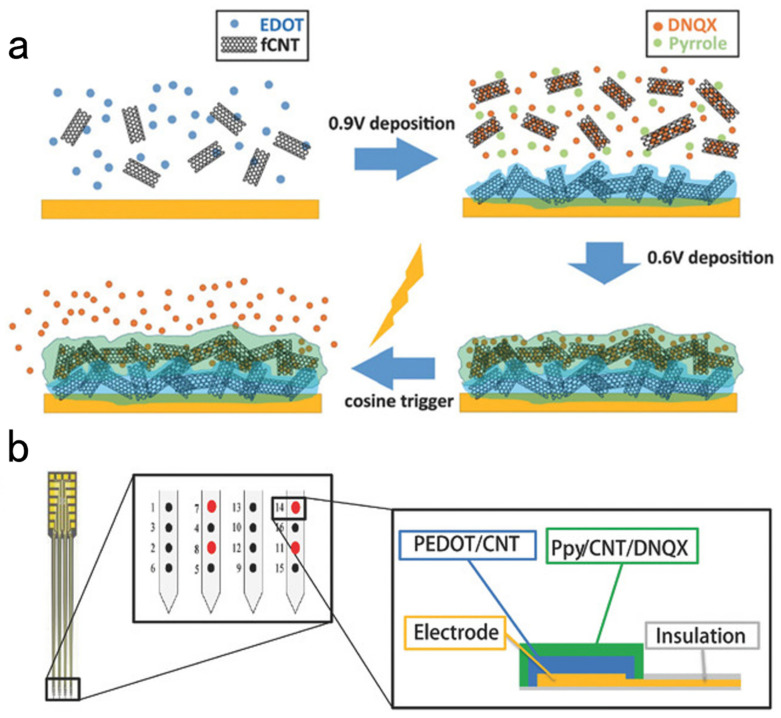
(**a**) Synthesis of the PEDOT/fCNT-PPy/fCNT/DNQX double-layer membrane and controlled release of DNQX from the membrane. (**b**) Dual-layer coating design of MEA in vivo.

Parkinson’s disease (PD) could be involved in the basal ganglion circuit. It consists of the substantia nigra (SN), striatum, subthalamic nucleus (STN), globus pallidus (GPi), cortex, etc. Deep brain stimulation (DBS) for STN and GPi is one of the most effective ways to treat dyskinesia symptoms. However, some researchers showed that different stimulation frequencies lead to different treatment outcomes. The mechanism of Parkinson’s disease, DA monitoring, and spike discharges under deep brain stimulation in rats with Parkinson’s disease was investigated. Therefore, Cai et al. [108] made an MEA with a length of 7 mm, a width of 250 μm, and a site modified with platinum nano-particles and reduced graphene oxide nanocomposites (Pt/rGO) by electroplating. It monitors DA concentration and nerve spike discharge in the caudate-putamen (CPU) of Parkinson’s disease rats in real-time. After DBS was applied to the medial pallidum (GPi) side of the PD rats, the electrode array detected a significant elevation in DA concentration in bilateral CPUs. The average increment of DA on the same side was 7.33 μM. The increment of DA on the comparison side was 2.2 times higher. The average amplitude of nerve spikes in both CPUs was reduced by more than 10%. The spike discharge rate was reduced by 65% (ipsilateral) and 51% (contralateral). It could be observed that DBS plays a vital role in regulating DA concentration, peak discharge, and the power of bilateral CPUs. In addition, the same side change of dual mode signal is more significant than the side. These results provide us with new detection and stimulation techniques to decipher the potential mechanism of Parkinson’s disease. Specific neuron discharge and DA neurotransmitters during STN-DBS were detected by further research. Similar spike-wave and DA concentration changes were detected when the simulation frequency ranged from 10 to 350 Hz. Moreover, it showed the highest spike-wave discharge frequency and DA concentration at around 100Hz of the stimulation frequency. Stimulation dramatically regulated patterns of MSNs, whereas FSI did not. Thus, the diverse neural spike wave modes have a distinct part in PD animals [110]. Silicon-based microelectrode arrays can regulate neural activity by controlling the release of neurochemicals from the coating of the microelectrode array sites and directly applying electrical stimulation to the corresponding neurons. This is an exciting function. For instance, Du et al. [111] developed a new double-layer conductive polymer/acid functionalized carbon nanotube microelectrode coating. It is applied to the classic Neuronexus 16 channel in vivo MEA. Moreover, it can load and electrically release the neurochemical 6,7-dinitroquinoline-2,3-dione (DNQX). DNQX is a 2-amino-3-(5-methyl-3-oxo-1,2-oxazol-4-yl) propionic acid (AMPA) receptor antagonist. AMPA receptor-mediated rapid excitability transfer directly affects neural network activities as it participates in generating action potentials. Therefore, releasing AMPA receptor antagonist DNQX triggered by electricity from the microelectrode coating could regulate the activity of neurons. The electrode coating prepared by this double-layer method had an inner layer of PEDOT/fCNT and an outer layer of PPy/fCNT/DNQX. The former enhances the impedance increase due to drug loading on the coating. In contrast, the latter is designed to improve drug loading. In addition, the mechanical stability of the double-layer coating can withstand surgical insertion and repeated in vivo drug release. The illustration of the synthesis of dual-layer film and the bilayer coating scheme on MEA in vivo is shown in Figure 10.

Cai et al. [112] designed a modified MEA with single-walled carbon nanotubes/PEDOT: PSS nanocomposites to optimize the electrochemical and electrical performance of MEA. Poly (3,4-ethylene dioxythiophene)/polystyrene sulfonate (PEDOT: PSS) has a porous structure with good adhesion. It has been widely used for electrophysiological signals and electrochemical detection [113,114,115]. Carbon nanotubes (CNTs) have also been used for modifying electrodes due to their excellent electrochemical performance, biocompatibility, and chemical stability [75,116]. Carbon nanotubes are embedded in PEDOT: PSS to enhance the conductivity, biocompatibility, and stability of MEA. The modified electrode had an electrical performance of 16.20 ± 1.68 kΩ low impedance and −27.76 ± 0.82° small phase delay, which enabled MEA to detect spikes with high SNR (>3). Regarding the electrochemical performance of dopamine detection, it showed low oxidation potential, high sensitivity, and a wide linear range.

### 4.3. Ceramic-Based MEAs

**Figure 11:**
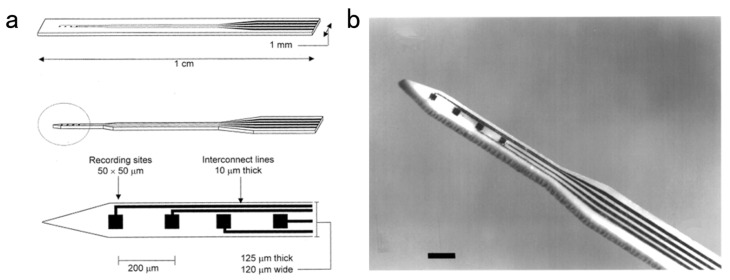
(**a**) Schematic diagram of the rectangular and tapered multi-site and ceramic-based microelectrodes. (**b**) A multi-site ceramic-based microelectrode tip.

**Figure 12:**
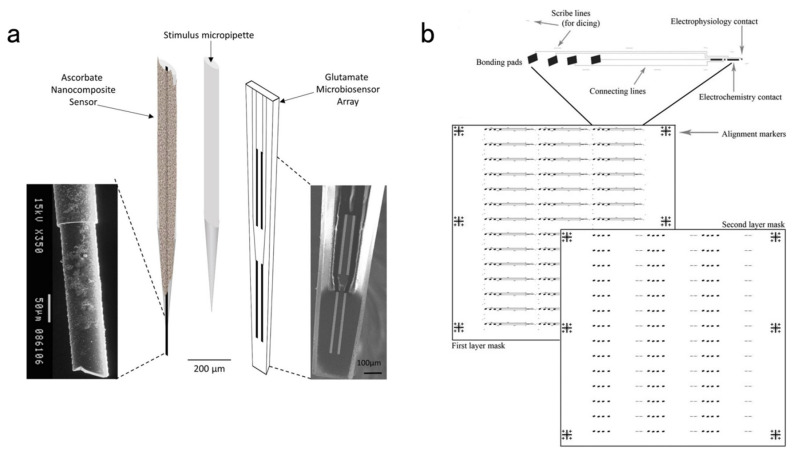
(**a**) The schematic diagram of the array of the ascorbate nanocomposite sensor (left), the stimulus micropipette (center), and the glutamate sensor (right) [120]. (**b**) The recording location circuit and the photolithographic mask [121].

The ceramic material is robust and easy to implant. It is a good material for use as an implantable microelectrode and an insulator used as a substrate for novel microelectrodes for reducing crosstalk between adjacent detecting sites [117]. GA Gerhardt et al. [118] developed a ceramic-based MEA by photolithography. The recording sites and connecting wires were made of platinum, and a polyimide coating insulated the connecting wires. A 1 cm-long microelectrode is cut from the wafer, gradually thinning with a tip of 2–5 μm. The schematic diagram of the multi-site ceramic-based microelectrode and the photograph of the tip is shown in Figure 11. Electrochemical detection of hydrogen peroxide and dopamine demonstrated that the selectivity, sensitivity, and response characteristics of the electrode surpass the previous silicon electrodes. This is the first demonstration of a microelectrode array manufactured by the ceramic substrate. The data support the hypothesis that these microelectrode arrays could be available for diverse electrophysiological and neurochemical detection. Through continuous exploration, GA Gerhardt et al. [119] proposed a new method for measuring extracellular γ-aminobutyric acid (GABA) and glutamate in vivo using ceramic-based MEA. This was also a new method to quantify GABA levels in vivo. A dual-enzyme reaction system used ceramic-based MEA consisting of GABA enzyme and glutamate oxidase (GluOx) to quantify GABA and glutamate. The endogenous glutamate was subtracted from the mixed signal of GABA and glutamate to develop pure GABA concentrations. Preliminary research in vivo and in vitro manifested that the novel MEA manufacturing could be a feasible tool for the joint determination of GABA and glutamate within the central nervous system (CNS). Nuno R. Ferreira et al. [120] designed the nanocomposite sensors. They comprised carbon fiber microelectrodes disposed of with nafion, carbon nanotubes, and ceramic-based microelectrode biosensor arrays. Those could detect ascorbate and glutamate in the brain with high temporal and spatial resolution and chemical sensitivity, as shown in Figure 12a. The nanocomposite sensor indicates the electrocatalytic characteristics of ascorbate oxidation. Compared with Ag/AgCl, it has a negative shift from +0.20 V to −0.05 V with a significant increase in the electroactive surface area. The glutamate sensor arrays revealed a high sensitivity of 5.3 ± 0.8 pA μM^−1^, a low LOD of 204 ± 32 nM, and a high selectivity against primary interfering substances. The dynamic interaction of ascorbate and glutamate was revealed by real-time and simultaneous detection in the hippocampus of anesthetized rats after local stimulation using KCl or glutamate. 

Anita A. Disney et al. [121] developed a ceramic-based multi-site recording array using photolithography. This contains two electrochemical detecting sites designed for parallel channel or reference (300 μm× 15 μm) with two electrophysiological detecting sites (15 μm× 15 μm). The recording location circuit and photolithographic mask are represented in Figure 12b. This system allows the simultaneous recording of in vivo electrochemical and electrophysiological signals. Non-concurrent in vivo detection of extracellular choline concentration and LFPs were used to demonstrate the natural movement between various arousal states in animals.

## 5. Conclusions

Effective collection and analysis of spike sequence data with chemical signals from multiple sites of the electrode array could help researchers analyze how neuronal groups collaborate and determine the function of specific brain regions. The reliable dual-mode electrode arrays for electrophysiological and electrochemical detection possess biomedical applications. We could study the relationship between neuroelectric and neurochemical activities and understand the correlation between different neuronal activities. Furthermore, synchronous recording from multiple neurons can assess the relationship between patterns of group behavior and activity, perception, cognition and sensory process patterns. 

The appearance of MEMS improves the reproducibility and standardization of silicon-based electrode manufacturing. However, it cannot be used on flexible electrodes. Although rigid-material-based electrode has good stability and processability, their damage to tissues cannot be ignored. The flexible-material-based electrode is a significant direction of our efforts. Continuous improvement is required in chronic stability for flexible-material-based electrodes, interface compatibility with multiple structures, and multi-mode detection. It is easy to choose materials that meet one or several properties. However, the tradeoffs in other aspects of electrode performance are often unavoidable. For instance, many experiments have established that carbon fiber electrode is an excellent electrochemical sensing tool. However, its fabrication process should be further programmed to promote the repeatability and stability of the electrode. Flexible electrodes significantly reduce the damage to organisms and the occurrence of immune rejection. However, whether most flexible electrodes can support long-term in vivo, chronic, and stable acquisition of signals remains to be investigated. Each part of the brain has its function but is connected through the signal pathway. Specific requirements are put forward for the hardware design and manufacturing process of electrodes to simultaneously monitor the electrochemical and electrophysiological signals in different brain regions. The appearance of dual-mode electrodes and the continuous optimization of their performance provide a powerful tool to study brain function, the mechanism behind physiological and pathological events, and the treatment of nervous system diseases through external intervention. 

## Figures and Tables

**Table 1:** A summary of various electrodes based on the electrode materials.

Electrode Material	Electrical Property	Type of Electrode	Others	Reference
PEDOT nanotube	Impedance:17 kΩ (before implantation); 87 kΩ (after implantation); 2.21 ± 0.7 MΩ (6–8 days after implantation)	Si-based electrode; PEDOT nanotube is deposited on the record sites	Targeting: barrel cortex	[63]
PEDOT/PSS	Conductivity: 155 S/cm; Impedance:7.4 kΩ	3D print for Pure Polymeric Electrode	Targeting: primary motor cortex (M1); HTU:12 dB	[59]
PEDOT film	Impedance ≈ 20 kΩ (Φ30 μm); Specific capacitance: 3.6 mF/cm^2^	PEDOT/CNT is coated on the Au sites	-	[64]
PEDOT/LiClO_4_	Impedance: 9 kΩ (1250 μm^2^)	Single and four-shank neural probes; Au sites	-	[65]
PPy nanotube	Impedance: 80 kΩ (1250 μm^2^)	Eight-channel Si substrate acute probe; Au sites	-	[66]
PPy/peptide	Impedance: 500–1700 kΩ	Michigan electrode; Au sites	Targeting: inferior colliculus or auditory Cortex; Coatings establish strong connection with the neural structure	[67]
PPy: PTSA 1:2 nanowire	Conductivity: 800 S/cm; CIC:67.1 mC/cm^2^ at 50 mV/s	PDMS substrate	Targeting: visual cortex for normal and epileptic rat; Yang’s modulus: 1.9 MPa	[68]
PPy/GO	Impedance: 26 kΩ (Φ50 μm); Charge Capacity Density: 278.83 mC/cm^2^ at 50 mV/s	Silicon substrate and Pt sites	-	[69]
PPy film	Impedance: 115 kΩ (Φ50 μm); Charge Capacity Density:190.98 mC/cm^2^ at 50 mV/s	Silicon substrate and Pt sites	-	[69]
PPy/PSS	Impedance: 30 kΩ (1250 μm^2^)	-	-	[70]
PPy/SLPE	Impedance: 390 kΩ (3900 μm^2^)	-	-	[71]
AuPt alloy nanoparticles	Impedance: 230 kΩ (2870 µm^2^)	Michigan electrode	Targeting: lateral globus pallidus	[72,73]
AuNPs	Impedance: 900 kΩ (2870 µm^2^)	Michigan electrode	SNR = 3.4	[73]
Pt	Impedance: 5 kΩ (Φ33 μm); CSC: 50–100 μC/cm^2^	Sixteen electrodes of 33 μm-diameter arranged in two rows of 8 electrodes	-	[74]
CNT/Au	Impedance: 38 kΩ (Φ20 μm)	32 electrodes with CNT/Au composite	Targeting: motor cortex from rat; cortical area V4 from monkey	[75]
CNT/PPy	Impedance: 770 kΩ; Capacitance: 755 mF/cm^2^	MEA	Targeting: motor cortex from rat; cortical area V4 from monkey	[75]
TiN	CIC: 4.45 mC/cm^2^	MEA	-	[76,77]
RGO fiber	CIC: 14 mC/cm^2^	MEA	Targeting: feline visual cortex	[78]
PI	Impedance: 59 kΩ (6400 µm^2^); CSC: 154 μC/cm^2^	2 mm long array with 14 TiN square-shaped microelectrodes	-	[77]

## Data Availability

Not applicable.
